# Lung Allograft Epithelium DNA Methylation Age Is Associated With Graft Chronologic Age and Primary Graft Dysfunction

**DOI:** 10.3389/fimmu.2021.704172

**Published:** 2021-10-07

**Authors:** Daniel T. Dugger, Daniel R. Calabrese, Ying Gao, Fred Deiter, Tasha Tsao, Julia Maheshwari, Steven R. Hays, Lorriana Leard, Mary Ellen Kleinhenz, Rupal Shah, Jeff Golden, Jasleen Kukreja, Erin D. Gordon, Jonathan P. Singer, John R. Greenland

**Affiliations:** ^1^ Pulmonary, Critical Care, Allergy and Sleep Medicine Division, Department of Medicine, University of California, San Francisco, San Francisco, CA, United States; ^2^ Medical Service, Veterans Affairs Health Care System, San Francisco, CA, United States; ^3^ Department of Surgery, University of California at San Francisco, San Francisco, CA, United States

**Keywords:** lung, allograft, epigenetic, aging, primary graft dysfunction (PGD)

## Abstract

Advanced donor age is a risk factor for poor survival following lung transplantation. However, recent work identifying epigenetic determinants of aging has shown that biologic age may not always reflect chronologic age and that stressors can accelerate biologic aging. We hypothesized that lung allografts that experienced primary graft dysfunction (PGD), characterized by poor oxygenation in the first three post-transplant days, would have increased biologic age. We cultured airway epithelial cells isolated by transbronchial brush at 1-year bronchoscopies from 13 subjects with severe PGD and 15 controls matched on age and transplant indication. We measured epigenetic age using the Horvath epigenetic clock. Linear models were used to determine the association of airway epigenetic age with chronologic ages and PGD status, adjusted for recipient PGD risk factors. Survival models assessed the association with chronic lung allograft dysfunction (CLAD) or death. Distributions of promoter methylation within pathways were compared between groups. DNA methyltransferase (DNMT) activity was quantified in airway epithelial cells under hypoxic or normoxic conditions. Airway epigenetic age appeared younger but was strongly associated with the age of the allograft (slope 0.38 per year, 95% CI 0.27–0.48). There was no correlation between epigenetic age and recipient age (P = 0.96). Epigenetic age was 6.5 years greater (95% CI 1.7–11.2) in subjects who had experienced PGD, and this effect remained significant after adjusting for donor and recipient characteristics (P = 0.03). Epigenetic age was not associated with CLAD-free survival risk (P = 0.11). Analysis of differential methylation of promoters of key biologic pathways revealed hypomethylation in regions related to hypoxia, inflammation, and metabolism-associated pathways. Accordingly, airway epithelial cells cultured in hypoxic conditions showed suppressed DNMT activity. While airway methylation age was primarily determined by donor chronologic age, early injury in the form of PGD was associated with increased allograft epigenetic age. These data show how PGD might suppress key promoter methylation resulting in long-term impacts on the allograft.

## Introduction

Advanced donor age increases the risk of allograft failure in most types of solid organ transplantations (1). In lung transplantation, overall survival rates in the first 3 years are reduced when allograft donors are over 60 years old (2, 3). Long-term survival following lung transplantation is worse than for other solid organs and is limited by chronic lung allograft dysfunction (CLAD), a clinical syndrome of progressive loss of lung function and fibrosis. CLAD risk is similarly increased when the lung donor is advanced in age (4). In addition, the association of donor telomere dysfunction with CLAD suggests this susceptibility may relate to accelerated aging of the lung allograft (5).

Biological aging is associated with DNA methylation patterns. Cytosine-phosphate-guanine (CpG) methylation in gene promoters and other regions selectively suppresses transcription, thus influencing cell phenotypes. Chronological age is another major driver of these DNA methylation patterns. Horvath described an epigenetic clock based on 353 representative CpG sites that are hyper- or hypomethylated in association with age across multiple tissue types (6). This epigenetic clock is thought to be driven by the epigenetic maintenance system, in which DNA methyltransferases (DNMTs) maintain CpG methylation and compete with active demethylation driven by ten-eleven translocation (TET) methylcytosine dioxygenases and passive demethylation (6, 7). These processes are reversed during embryogenesis, when the epigenetic clock is reset (8). Importantly, epigenetic aging can be accelerated by stressors such as cancer, HIV infection, metabolic syndromes, and Alzheimer’s disease (6, 9). Conversely, super-centenarians and their offspring have been shown to have “younger” epigenomes (10). DNMT activity is also targetable with existing drugs that could potentially influence aging phenotypes and longevity (11).

Early events following lung transplantation influence CLAD risk and long-term allograft survival. Primary graft dysfunction (PGD), a form of ischemia-reperfusion injury, results in airway damage and cell turnover and is an independent risk factor for CLAD development (12). PGD may contribute to CLAD through enhanced alloimmune activation or by rendering the allograft less resilient to ongoing stress, as might be seen with aging. However, it is not known whether lung allograft epigenetic age is determined by donor or recipient birth dates nor how allograft stressors impact epigenetic ageing. Here, we hypothesized that lung allograft epigenetic age is determined by the donor age and would be accelerated by the stress of severe PGD.

## Material and Methods

### Human Subjects

We selected lung transplant recipients from an ongoing longitudinal cohort at the University of California San Francisco. Lung transplant candidates were approached for study enrollment at the time of listing or as soon as possible following lung transplant. All subjects provided informed consent for medical record review and research airway brushing during bronchoscopy (UCSF IRB 13-10738). From these consented subjects, we selected a case-control cohort for analysis of DNA methylation patterns in cultured airway epithelial cells.

Severe PGD was scored by two transplant pulmonologists based upon retrospective chart review and was defined as grade 3 (PaO2:FiO2 < 200) at 48–72 h post-transplant according to international criteria (13). Subjects with severe PGD were considered for inclusion if 1-year brushings yielded epithelial cell cultures, they had bilateral lung transplant, and the allograft donor had no tobacco-use history. Control subjects, who had PGD grade ≤1 through 72 h post-transplant and 1-year brushing airway epithelial cell cultures, were frequency matched to cases based upon recipient age and transplant indication.

Time to CLAD was defined as the days after transplant to the first forced expiratory volume in 1 s (FEV_1_) value observed to be ≤20% decreased from post-transplant baseline, after exclusion of other causes of FEV_1_ decline, such as pleural effusions, per 2019 International Society of Heart and Lung Transplantation consensus guidelines (14). For this study, we did not differentiate between obstructive and restrictive forms of CLAD.

Lung transplantation induction and maintenance immunosuppression regimens followed previously described institutional protocols (15), with basiliximab and methylprednisolone induction. During the first 3 months, prednisone was dosed at 20 mg daily and tacrolimus dosed to 10 to 14 ng/ml trough concentrations. For months 3–6, prednisone was tapered to 0.2 mg/kg daily and tacrolimus targeted 10 to 12 ng/ml. Prednisone was then tapered to 0.1 mg/kg by 12 months and tacrolimus adjusted to 8 to 10 ng/ml. Mycophenolate mofetil targeting 2 g daily in divided doses was started postoperatively. To prevent neutrophilic reversible allograft dysfunction, 250 mg of azithromycin three times weekly is initiated 30 days after the transplant surgery. Immunosuppression was tailored as clinically indicated.

### Airway Brushing and Epithelial Cell Culture

Small airway brushing was performed in the distal airways as part of routine 1-year post-transplant surveillance bronchoscopy (16). Following bronchoalveolar lavage (BAL) and before transbronchial biopsies, a cytology brush (Conmed #129) was advanced under fluoroscopic guidance into a basilar segment airway to about 3–4 cm from the periphery. The brush was agitated approximately 10 times and pulled back into the catheter. The brush tip was then transferred to a centrifuge tube containing Roswell Park Memorial Institute (RPMI) 1640 media. The tube was vortexed gently to dislodge cells from brush then centrifuged. The media was decanted from cell pellet and an aliquot of 0.25% trypsin used to separate epithelial cell clumps. Trypsin was neutralized with fetal bovine serum (FBS), then the tube was centrifuged again. The cell pellet was then resuspended in airway epithelial growth media containing 5% FBS and 10 uM rho kinase inhibitor (Selleck Chem, Y-27632). Cells were plated into sterile cultured-treated 10 cm dishes that were coated in rat tail collagen type I. The dishes were cultured in 37°C and 5% CO_2_ incubator with media change every 48 h until 80% confluent. The media was removed and the plates washed with warm PBS. Trypsin 0.25% was used to liberate epithelial cells from the plates then neutralized with 5% FBS-containing growth media. Cells were collected and centrifuged and resuspended in 50% FBS, 10% DMSO, and 40% growth media, then cryopreserved. The cells were stored in the vapor phase of liquid nitrogen until use.

### Airway Epithelial Cell Flow Cytometry Analysis

Cultured airway cells were incubated with Human TruStain FcX (BioLegend 422302) to block non-specific binding and Fixable Viability Dye eFluor 660 (Invitrogen 65-0864-14) as a dead cell marker. Cells were then stained with R-phycoerythrin-Cy7 anti-human CD45 (Invitrogen 25-0459-42, clone HI30) or R-phycoerythrin-Cy7 anti-human nerve growth factor receptor (NGFR, BioLegend 345110, clone ME20.4), Brilliant Violet 786 anti-human Epithelial cell adhesion molecule (EpCAM, BD Biosciences 565685, clone EBA-1), or Brilliant Violet 510 anti-human Carcinoembryonic antigen-related cell adhesion molecule 6 (CEACAM6, BD Biosciences 742684, clone B6.2). Flow data were acquired using the FACSAria Fusion Flow Cytometer (BD Biosciences) and analyzed using FlowJo Software. Negative population thresholds were set using unstained airway epithelial cells.

### DNA Methylation Age Analysis

Frozen cell vials were thawed in 37°C water bath. The cells were washed with fresh media and pelleted by centrifugation. Media was decanted from the pellet. Genomic DNA was isolated from the cell pellet following manufacturer directions for the GenElute Mammalian Genomic DNA Miniprep Kit (Sigma-Aldrich G1N70). Genomic DNA was quantified and checked for quality by NanoDrop. DNA methylation analysis was performed following manufacturer directions for Illumina 450K, Infinium methylationEPIC array. Methylation ages were determined using RnBead’s implementation of Horvath’s epigenetic clock. Here, elastic net regression was used to predict age from a training set of 1,866 samples from 20 studies of blood, kidney, and brain tissue, yielding 761 age-associated CpGs in the methylationEPIC 450K array (17). Due to a pandemic-related shutdown of a genomics core facility, Infinium arrays were processed in two separate facilities. RnBeads age calculations were performed separately on each batch.

Allograft chronologic age was defined as date of airway brush minus donors’ birthdates.

### Hypoxia and DNA Methyltransferase Activity

We grew the human bronchial epithelial cell line 16HBE14o- (Sigma-Aldrich, SCC150) in sterile tissue-culture treated plates. At 80–90% confluence, cells were exposed to 1% O_2_ and 5% CO_2_ or 21% O_2_ and 5% CO_2_ in humidified 37°C incubators for 24 and 48 h. Cells were washed with PBS and collected by 0.25% trypsin-EDTA followed by trypsin neutralization with FBS, centrifugation, and enumeration. The Epigentek Nuclear Extraction Kit (OP-0002-1) was used to isolate nuclear proteins. Protein concentration was determined by bicinchoninic acid (BCA) assay (Thermo Fisher) and NanoDrop technology. Ten micrograms of nuclear extract protein were used in the DNMT (DNA methyltransferase) activity assay (Epigentek P-3009) or TET (ten-eleven translocation enzyme) activity assay (P-3086) following the manufacturer’s protocols to determine DNMT and TET activity with or without exposure to hypoxia.

### Statistical Analysis

Subject characteristics between cases and controls were compared by chi-squared test for categorical variables. Shapiro-Wilk normality test was used to determine subsequent statistical testing for continuous variables in the subject characteristics table. Student’s t-test was used for normally distributed continuous variables and Mann-Whitney U test for non-parametric continuous variables (“tableone” package, R version 4.0.3, R Foundation for Statistical Computing, Vienna, Austria). Mean and standard deviation are shown for normal data or median and interquartile range shown for non-normal data throughout.

The associations between methylation age and graft age were determined using Pearson’s product-moment correlation and univariable linear regression. The impact of PGD status on observed methylation age was assessed by Mann-Whitney U-test and multivariable linear models. We sequentially added covariates starting with PGD as a predictor and including batch effect as a covariate. Subsequent multivariable models included (1) donor age; (2) model 1 plus donor sex, donor body mass index (BMI), donor cytomegalovirus (CMV) status, and donor White ethnicity; (3) model 2 plus recipient age, recipient sex, recipient BMI, recipient White ethnicity, and transplant indication. Model fits were compared by analysis of variance tables (ANOVA).

Time to CLAD or death association with airway methylation age was determined by Cox proportional hazards model using the “survival” R package. This model was left truncated at the time of airway brush and included tertile of methylation age as a categorical predictor variable and batch effect as a covariate. Power analysis was performed by simulating predictor variable distributions and fitting a logistic regression curve to the outcome of P<0.05 for a survival model using the observed CLAD-free survival times as a function of simulated hazards ratio. Kaplan–Meier plots were generated using the R “survminer” library.

Average genome-wide CpG methylation was computed in RnBeads in each batch and compared across groups using Student’s t-test to examine for global differences in methylation status. To examine differential promoter methylation, CpG sites were aligned to gene promoters in RnBeads. Promoter methylation frequencies were normalized within batches prior to pooling. Differential methylation was assessed by Student’s t-test using the R “viper” library and adjusted for multiple comparisons using the Benjamini–Hochberg method. To examine differential methylation within specific pathways, we examined promoters for genes in MSigDB Hallmark curated gene sets (18). T-statistic distribution for differential methylation across gene sets was assessed by Kolmogorov–Smirnov tests for each pathway, with p-values adjusted with the Benjamini–Hochberg method, as previously described (19). The top 15 most differentially methylated pathways with FDR P-values <0.01 are shown.

## Results

Airway cell cultures were performed using established protocols that have been previously shown to yield pure epithelial cell populations in studies of asthma (20). We performed flow cytometry to verify that these samples collected from transplant recipients were indeed pure airway epithelial cell populations. In a representative set of airway epithelial cells cultured at 1 year from five subjects, 99% (range 98.2–99.4%) were EpCAM positive epithelial cells, with 0% (range 0–0.2%) CD45+ immune cells. We further characterized these cells as basal or secretory using NFGR and CEACAM6 antibodies, as previously published (21). These cells included 59.7% NGFR+ basal cells (range 30.5–73.2%) and 22% CEACAM6+ secretory cells (range 14.1–30.8).

We assessed DNA methylation (DNAm) age 1 year after transplantation in allograft airway epithelial cells. Thirteen subjects with severe PGD were matched with 15 subjects without PGD within 72 h of lung transplantation. Subject characteristics for these groups are shown in **Table 1**. Stratified by PGD status, subject characteristics did not differ between these groups. Specifically, there were no differences between recipient ages (57.0 *vs.* 60.5, p = 0.34), CMV risk group (p=0.55), HLA mismatches (4 *vs.* 4, p = 0.22), recipient sex (p = 0.24), donor sex (p=1), and Lung Allocation Scores (49.3 *vs.* 56.0, p = 0.52). The donor age trended higher in the PGD group, but this difference did not reach statistical significance (33.7 *vs.* 43.6, p = 0.08). There was no difference in the time it took from cells to expand in culture between the two groups (p = 0.28).

**Table 1:** Subject characteristics.

N	Non-PGD	PGD	p-value
	15	13	
**Recipient age**, median [IQR]*	57.0 [26.8, 65.0]	60.5 [55.0, 63.5]	0.34
**Male recipient**, N (%**)**	6 (40)	9 (69)	0.24
**Donor age**, mean (SD)	33.7 (11)	43.6 (17)	0.08
**Male donor**, N (%)	11 (73)	10 (77)	1
**Recipient BMI,** mean (SD)	23.9 (5.0)	28.3 (3.6)	0.01
**Donor BMI,** mean (SD)	23.2 (4.1)	25.7 (3.3)	0.09
**Recipient ethnicity**, N (%)			0.21
American Indian/Alaska Native	0 (0)	2 (15)	
Asian	2 (13)	3 (23)	
Black	2 (13)	0 (0)	
White	11 (73)	8 (62)	
**Donor ethnicity**, N (%)			0.56
American Indian/Alaska Native	1 (8)	3 (23)	
Asian	4 (31)	2 (15)	
Black	2 (15)	1 (8)	
White	6 (46)	7 (54)	
**Transplant indication category**, N (%)			0.31
A (obstructive)	1 (7)	1 (8)	
B (pulmonary vascular)	0 (0)	0 (0)	
C (cystic fibrosis)	4 (27)	1 (8)	
D (restrictive)	10 (67)	11 (85)	
**CMV status**, N (%)			0.55
Donor (−) Recipient (−)	2 (13)	2 (15)	
Donor (−) Recipient (+)	3 (20)	4 (31)	
Donor (+) Recipient (−)	6 (40)	2 (15)	
Donor (+) Recipient (+)	4 (27)	5 (39)	
**HLA mismatches**, median [IQR]*	4 [4, 5]	4 [4, 6]	0.22
**Double lung transplant**, N (%)	15 (100)	13 (100)	1
**Post-transplant days**, median [IQR]*	370 [363, 371]	370 [369, 380]	0.24
**Days of airway cell culture**, median [IQR]*	14 [13, 19]	15 [14, 17]	0.28
**Lung allocation score**, median [IQR]*	49.3 [42.1, 62.8]	56.0 [44.6, 85.7]	0.52

BMI, body mass index in kg/m^2^; CMV, cytomegalovirus; HLA, human leukocyte antigen; IQR, interquartile range; N, number; PGD, severe primary graft dysfunction; SD, standard deviation

*Indicates a non-normal distribution was identified by Shapiro-Wilk test p-value of <0.05. These variables are reported as median with IQR and compared by Mann-Whitney U test.

**Figure 1:**
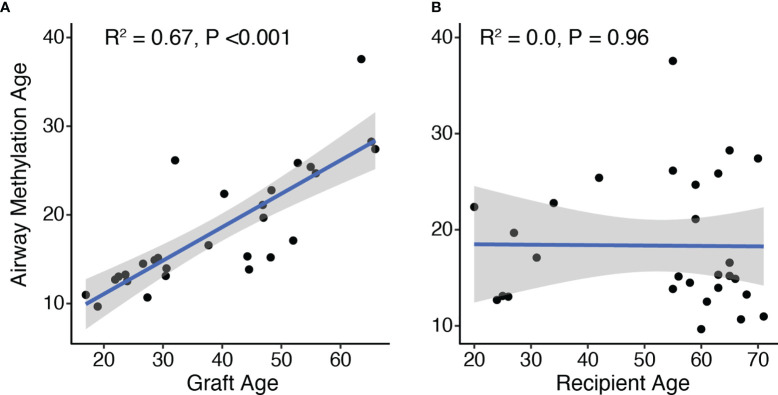
DNA methylation age correlates with allograft donor but not recipient ages. The age predicted by DNA methylation pattern was compared to the age of the graft **(A)** or recipients **(B)** based on time between airway brush and donor or recipient birthdate, respectively. R^2^ and p-values for the 28 subjects were determined by Pierson’s product moment correlation. By linear regression, airway methylation age increased by 0.38 (95% CI 0.27–0.48) per year of allograft age.

We examined predicted airway epithelial cell methylation age as a function of graft and recipient age. Airway epithelial cell methylation was highly correlated with the age of the allograft (R^2 =^ 0.67, p<0.001; **Figure 1A**). In contrast, airway methylation age predictions were not correlated with recipients’ chronologic ages (**Figure 1B**). There was also no association between airway methylation age and transplant indication (P = 0.49 by Kruskal-Wallis test). Despite a strong correlation, airway methylation ages were generally less than half of the allograft age. The median ratio of methylation age to graft age was 0.37, with an interquartile range 0.23–0.53. A linear fit of graft methylation *versus* chronologic age had a slope of 0.38 (95% CI 0.27–0.48) per year of allograft age.

**Figure 2:**
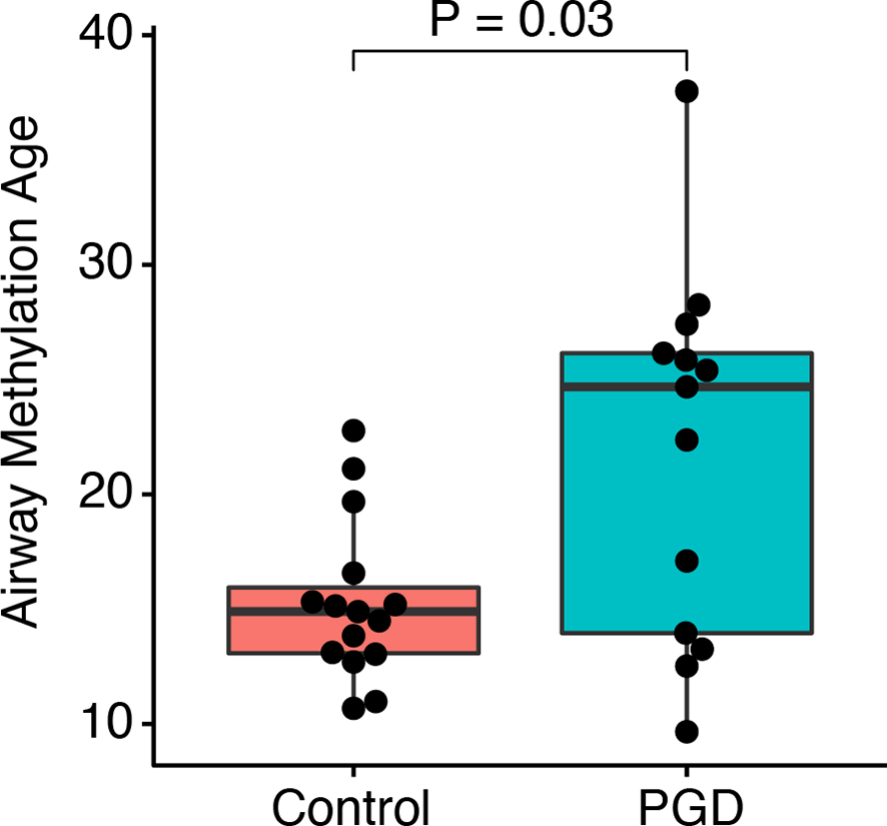
PGD is associated with accelerated DNA methylation age. DNA methylation age for non-PGD and PGD subjects was compared in airway cell cultures taken 12 months after lung transplantation. Groups were compared by Mann-Whitney U test. By linear regression, airway methylation age was 6.5 years (95% CI 1.7–11.2 years, P = 0.01) greater for n=15 PGD *versus* n=13 non-PGD subjects.

We then asked if PGD is associated with accelerated airway methylation aging. The mean airway methylation age was greater in the subjects who had experienced PGD as compared to controls (21.9 *versus* 15.3 years, Mann-Whitney U-test p = 0.03; **Figure 2**). In a regression model adjusting for batch effects alone, PGD was associated with a 6.5-year increase in methylation age (p = 0.01). Additional models adjusting for donor age or multiple factors (both recipient and donor characteristics) continued to show increased airway methylation age associated with PGD compared to airways without PGD (**Table 2**). Although donor age accounted for a significant proportion of the observed difference, airway methylation age was increased in the PGD group by 3.4 (95% CI 0.7–6.2) years, even after adjusting for the donor and recipient characteristics listed in **Table 2**. Using only donor age, donor BMI, and recipient BMI, which were the characteristics with a P-value <0.10 for difference between cases and controls, severe PGD was associated with a 3.9 (95% CI 0.6–7.2) year increase in airway epigenetic age.

**Table 2:** PGD effect in sequential regression models of airway methylation age.

Predictor variables	Residual sum of squares	Methylation age increase with PGD (95% CI)	p-value for PGD
**PGD**	938	6.5 (1.7–11.2)	0.01
**+ donor age**	314*	3.6 (0.7–6.5)	0.02
**+ donor sex, BMI, CMV status**, **and White ethnicity**	222	3.6 (0.6–6.5)	0.03
**+ recipient age, sex, BMI, and White ethnicity**; **and transplant indication**	111	3.4 (0.7–6.2)	0.03

All models adjusted for batch effects. (*) denotes statistically significant improvement in model fit. 95% CI, 95% confidence intervals; BMI, body mass index; CMV, cytomegalovirus; HLA, human leukocyte antigen; PGD, severe primary graft dysfunction.

**Figure 3:**
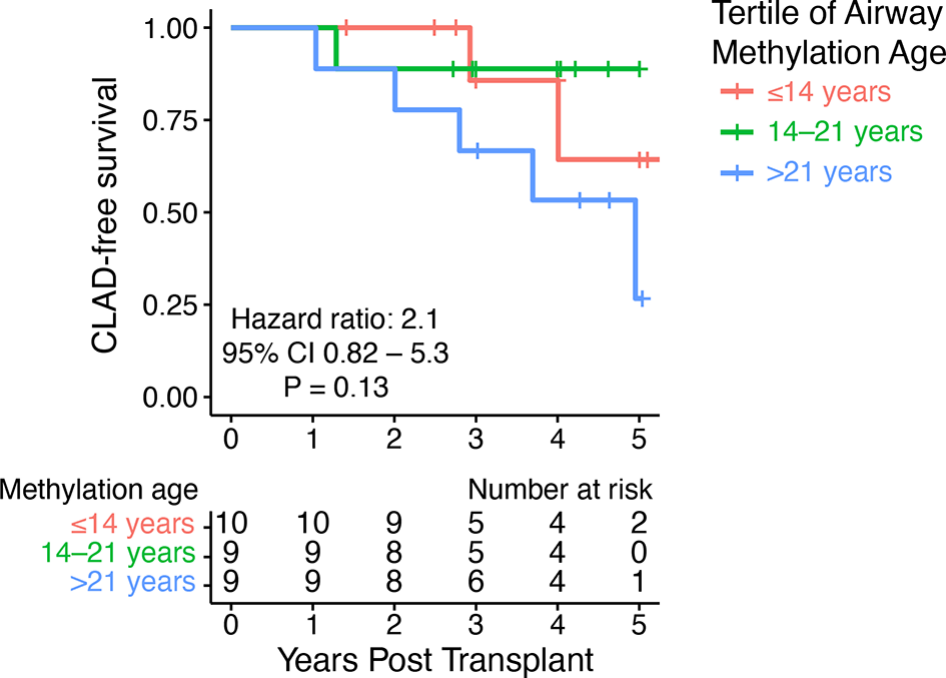
DNA methylation age did not predict CLAD-free survival. A Kaplan–Meier plot shows time to CLAD or death stratified by tertiles of airway methylation age. The hazard ratio for CLAD or death was 2.1 (95% CI 0.82–5.3) per tertile of airway methylation age determined by Cox proportional hazards modeling.

To examine the association between donor methylation age and CLAD-free survival, we performed Cox proportional hazards modeling. We observed a 1.05 (95% CI 0.97–1.14) hazard of CLAD or death per unit increase in airway methylation age. Of note, the available outcome data provided only 3% power to detect a statistically significant difference of this magnitude. Addition of donor age to this model resulted in a hazard ratio of 1.12 (95%CI 0.94–1.34) per unit increase in airway methylation age. When subjects were divided into tertiles based upon youngest to oldest airway methylation age, we observed a trend toward decreased CLAD-free survival only in the group with the oldest DNA methylation age compared to the other two tertiles (HR 4.1, 95% CI 0.96–17.7, P = 0.056), but when each tertile was considered separately, there was no significant difference in CLAD-free survival (**Figure 3**).

**Figure 4:**
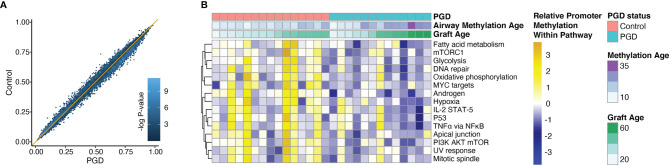
Promoters within specific gene sets were hypomethylated relative to non-PGD subjects. Average methylation values were determined for CpG sites in gene promoter regions. Average values for PGD subjects (x-axis) are plotted against average values non-PGD control subjects (y-axis) for each CpG site. There were no differentially methylated promoters after adjustment for multiple comparisons. Each dot color corresponds to the -log of the unadjusted p-value for each site **(A)**. Promoter methylation within the MSigDB Hallmark gene sets were compared for between PGD and control subjects. The top 15 most differentially methylated pathways are shown, all with a false discovery-adjusted P-value of <0.01 by Kolmogorov–Smirnov test **(B)**.

Given the observed differences in airway methylation age between PGD and non-PGD cohorts, we sought to determine if there were other effects of PGD on epithelial cell methylation patterns. We observed no difference in global average CpG methylation values between the two groups (mean genome-wide methylation frequency 0.47 *vs.* 0.47, p = 0.35). Similarly, when we examined the average methylation frequency of the 40,086 CpG sites within gene promoter regions, we found that PGD cases and controls were tightly correlated with no specific CpG site having a statistically significant difference in methylation after false-discovery adjustment (**Figure 4A**). Next, we sought to discover if there could be functionally important differences in methylation among key biological pathway promoters. Interestingly, no promoters within the Hallmark MSigDB gene sets were significantly *hyper*methylated in the PGD group relative to the referent group. However, there were several gene sets that were found to be *hypo*methylated in the PGD subjects relative to controls. Notably, promoters within gene sets related to TNFα, mTOR signaling, IL2-STAT5, and hypoxia were hypomethylated, changes that generally increase transcription, in the PGD group (**Figure 4B**).

**Figure 5:**
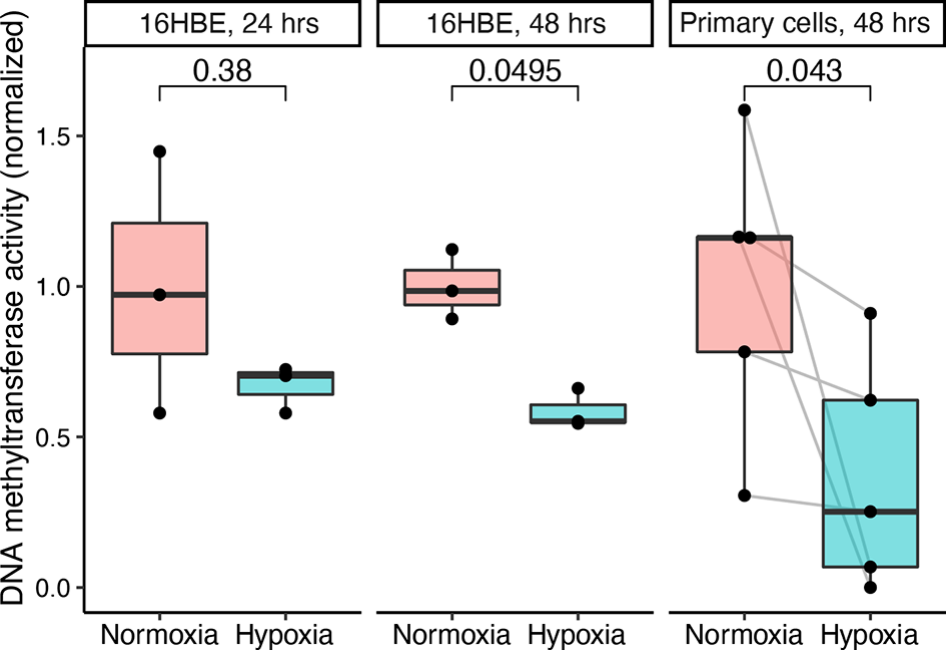
Hypoxia attenuates DNA methyltransferase (DNMT) activity in human bronchial epithelial cells. The human bronchial epithelial cell line 16HBE140- was cultured and exposed to 1 or 21% oxygen for 24 or 48 h. Similarly, primary epithelial cells cultured from small airway brushes were exposed to 1 or 21% oxygen for 48 h. DNMT activity was measured and normalized to the normoxia group mean values. Hypoxia and normoxia values were compared in 16HBE140- cells using the Wilcoxon rank sum test and in primary cells using the Wilcoxon signed rank test (paired by subject identifier).

To dissect the mechanism behind this finding, we examined the potential effects of hypoxia on airway epithelial cell methylation. We mimicked the hypoxia that is induced in allografts during the transplant procedure by exposing human bronchial epithelial cells to 1% oxygen before measuring DNMT activity. After 24 h of hypoxia, there was no significant difference in DNMT activity. However, 48 h of hypoxia revealed a nearly twofold suppression of DNMT activity in the hypoxic group (**Figure 5**). We also measured ten-eleven translocation (TET) enzyme activity, but no measurable activity was detected under either condition in those experiments.

## Discussion

In lung recipients with severe PGD, we found accelerated epigenetic aging of allograft epithelial cells obtained 1 year later. We also discovered significant hypomethylation of promoters within key gene sets in subjects with severe PGD as compared to matched subjects with no PGD. Finally, we used a human bronchial cell line *in vitro* to discern the effects of hypoxia on DNMT and TET activity and found DNMT activity was impaired by hypoxic culture conditions, an effect that could potentially drive hypomethylation of active promoter regions.

Epithelial cells sampled from lung allografts 12 months after transplantation demonstrate a DNA methylation age that was strongly correlated with donor chronologic age and independent of recipient age. While the degree of correlation was similar to what has been reported lung tissue using a multi-tissue epigenetic clock, here airway methylation ages were approximately half of expected ages (6). This difference likely reflects tissue-specific methylation patterns since the methylation age regression formula we used was primarily derived from peripheral blood tissue. Similar ratios between predicted and expected methylation age have been observed for brain and semen using peripheral blood-based epigenetic clocks (7, 17). As expected, airway methylation age did not correlate with the chronologic age of the graft recipient, suggesting that airway methylation age is not transferrable. Similarly, preservation of donor age is observed up to 17 years following hematopoietic stem cell (HSC) transplant (22), while human HSCs transplanted into humanized mice did not have the accelerated airway methylation aging patterns observed in mice (23). Together these findings support the theory that epigenetic clocks reflect cell-intrinsic effects of epigenetic maintenance activity over time.

PGD can result in profound epithelial cell necrosis and turnover (12). Thus, it is reasonable that severe PGD would accelerate airway methylation aging, as has been observed with other significant stressors (22). However, an alternative explanation is that advanced airway methylation age preceded PDG or even contributed to PGD pathogenesis. A longitudinal assessment of airway methylation age starting pre-transplant would be needed to address this possibility. Also, PGD may predispose to other events within the first year, such as infection or rejection, that could mediate accelerated aging. Another possibility is that stress and inflammation from PGD could drive again both within the allograft and peripherally. Further studies will be needed to assess the impacts of PGD and transplant on epigenetic age measured in peripheral blood cells. In addition, we did not find that airway methylation age predicted with subsequent clinical pathology in this study. While differential methylation analysis suggested increased activation of potentially pathologic pathways, additional work is needed to discern if advanced airway methylation age corresponds to a detrimental phenotype that may warrant a specific intervention.

Using the publicly available MSigDB Hallmark gene sets, we identified a discrete list of pathways for which promoters were relatively hypomethylated in the severe PGD group. We also found DNMT activity to be suppressed by hypoxic culture conditions in a human bronchial epithelial line. DNMT activity has been shown to be important for preventing hypomethylation in regions of active transcription (24), so the pathways observed to be hypomethylated when DNMT activity was suppressed by hypoxia may be those most activated in the context of severe PGD. As PGD is an ischemia-reperfusion injury, hypomethylation of hypoxia-related gene promoters is perhaps the least surprising. Indeed, hypoxia following PGD could potentially persist well beyond the 72 h of the study definition secondary to organizing diffuse alveolar damage or vascular dysfunction (25). Glycolytic gene promoters, an oxygen-independent metabolic pathway, were also found to be hypomethylated in subjects that experienced PGD, as were inflammatory and cell cycle pathways. Increased activation of these pathways secondary to promoter hypomethylation could be one mechanism linking PGD and CLAD. Further studies are needed to determine whether therapeutic interventions during PGD with small molecule modulators of these pathways could have long-term impacts on gene expression.

This study had some important limitations. It included the use of a relatively small, single-center cohort, and so the findings need validation in other centers where surgical or clinical protocols might differ. It was underpowered to detect small to moderate effects of DNA methylation age on CLAD-free survival. We did not determine how hypoxia leads to DNMT suppression. In cardiac tissue, expression of DNMT enzymes is influenced by hypoxia-inducible factor (HIF)-1α, but this may not apply in lung cells (26). We were also unable to measure significant activity of TET enzymes that are responsible for DNA demethylation. While these enzymes require oxygen as a cofactor for their activity, hypoxic effects on TET activity are reported to be cell type dependent. Minimal impacts of hypoxia on TET activity were reported in the A549 alveolar epithelial cells, consistent with our observations (27, 28).

Using cultured epithelium for the methylation analysis *versus* a sample taken immediately from the airways may yield different findings. Prior to culture, airway brushes may contain up to 45% immune cells (29). Because immune cells have significantly greater epigenetic age at baseline, the impacts of cell type composition would likely overwhelm any signal from epigenetic aging. Based on our flow cytometry analyses and prior studies, these cultured epithelial cells were very unlikely to have significant immune cell contamination (20). If there were an effect from time in cell culture or immune cell contamination, these would likely bias our results towards the null. However, it is possible that differences in epithelial cell subtype distributions may affect the observed airway epigenetic ages. Such differences might reflect increased senescence from cell turnover related to primary graft dysfunction. Future studies will be needed to investigate potential impacts of PGD on the distribution of airway epithelial cell subtypes. However, we have found that airway epithelial cell phenotypes persist over several cell culture passages (19) and we cultured cells from PGD subjects identically to the control group to minimize any confounding issues related to *ex vivo* culture. Donors in the severe PGD group were also slightly older than for the non-PGD group. Multivariable modeling suggested that this age difference only explained part of the observed difference in methylation age associated with PGD, but because we cannot exclude the potential for residual confounding, future studies with equal donor ages between PGD cases and controls would be needed (30).

PGD is a risk factor for the development of chronic lung allograft dysfunction (CLAD), an important barrier to long-term allograft success. Telomere shortening is a feature of aging and contributes to pulmonary fibrosis pathologies that have similarities to CLAD. Indeed, allograft telomere dysfunction has been shown to be a risk factor for CLAD as well (31). At the same time, telomere dysfunction has been linked to methylation aging (32). Combined analysis of methylation aging, telomere length, and their sequelae may contribute to donor risk stratification and to understanding CLAD pathogenesis.

While the effects of ischemia on DNA methylation in lung allografts have not previously been reported, ischemia-related methylation changes have been described in the context of kidney allografts (33, 34). In a rat kidney model of ischemia-reperfusion, ischemia reduced methylation of a promoter associated with the IFN-γ response element in the C3 gene promoter (35). A second study confirmed this finding in a rat syngeneic kidney transplant model (36). We did not observe an impact of PGD on C3 gene promoter methylation, even in unadjusted analysis. However, the ways in which hypoxia impact DNA methylation may vary across organ types. Further, the phenotype of advanced epigenetic age is likely different in recipient and allograft cells (37).

The findings reported here suggest methylation aging could be an important tool in the monitoring allograft health. PGD in the lung allograft was associated with an increase in the epigenetic age, and older methylation age showed a trend toward poorer outcomes. Further, hypoxia impacts on methylation maintenance enzymes resulting in long-term changes in gene expression could potentially link early graft events to long-term outcomes.

## Data Availability Statement

The data presented in the study are deposited in the Mendeley Data repository: https://www.doi.org/10.17632/wvg88v67nb.1.

## Ethics Statement

The studies involving human participants were reviewed and approved by the Human Research Protection Program, University of California, San Francisco. The patients/participants provided their written informed consent to participate in this study.

## Author Contributions

JG and DD designed the study, analyzed data, and wrote the report. JS, DC, JM, and YG performed PGD phenotyping. SH, LL, MK, RS, JG, and JS performed airway brushes for research. EG oversaw airway brush culturing. FD and DD performed DNMT assays. TT performed flow cytometry. All authors contributed to the article and approved the submitted version.

## Funding

This work was funded by the Veteran Affairs Office of Research and Development, CSR&D section (CX002011, JG), the National Heart Lung and Blood Institute (HL151552, JG), the National Institute of Allergy and Infectious Diseases (AI136962, EG), and a Cystic Fibrosis Foundation grants (GREENL16XX0 and HAYS19AB3).

## Conflict of Interest

The authors declare that the research was conducted in the absence of any commercial or financial relationships that could be construed as a potential conflict of interest.

## Publisher’s Note

All claims expressed in this article are solely those of the authors and do not necessarily represent those of their affiliated organizations, or those of the publisher, the editors and the reviewers. Any product that may be evaluated in this article, or claim that may be made by its manufacturer, is not guaranteed or endorsed by the publisher.

## References

[1] Dayoub JC Cortese F Anžič A Grum T de Magalhães JP. The Effects of Donor Age on Organ Transplants: A Review and Implications for Aging Research. Exp Gerontol (2018) 110:230–40. doi: 10.1016/j.exger.2018.06.019 PMC6123500 29935294

[2] Bittle GJ Sanchez PG Kon ZN Claire Watkins A Rajagopal K Pierson RN. The Use of Lung Donors Older Than 55 Years: A Review of the United Network of Organ Sharing Database. J Heart Lung Transplant (2013) 32:760–8. doi: 10.1016/j.healun.2013.04.012 23664760

[3] Baldwin MR Peterson ER Easthausen I Quintanilla I Colago E Sonett JR. Donor Age and Early Graft Failure After Lung Transplantation: A Cohort Study. Am J Transplant (2013) 13:2685–95. doi: 10.1111/ajt.12428 PMC4157369 24034167

[4] Hennessy SA Hranjec T Swenson BR Kozower BD Jones DR Ailawadi G. Donor Factors are Associated With Bronchiolitis Obliterans Syndrome After Lung Transplantation. Ann Thorac Surg (2010) 89:1555–62. doi: 10.1016/j.athoracsur.2010.01.060 PMC3033807 20417777

[5] Naikawadi RP Green G Jones KD Achtar-Zadeh N Mieleszko JE Arnould I. Airway Epithelial Telomere Dysfunction Drives Remodeling Similar to Chronic Lung Allograft Dysfunction. Am J Respir Cell Mol Biol (2020) 63(4):490–501. doi: 10.1165/rcmb.2019-0374OC 32551854 PMC7528921

[6] Horvath S. DNA Methylation Age of Human Tissues and Cell Types. Genome Biol (2013) 14:R115. doi: 10.1186/gb-2013-14-10-r115 24138928 PMC4015143

[7] Day K Waite LL Thalacker-Mercer A West A Bamman MM Brooks JD. Differential DNA Methylation With Age Displays Both Common and Dynamic Features Across Human Tissues That are Influenced by CpG Landscape. Genome Biol (2013) 14:R102. doi: 10.1186/gb-2013-14-9-r102 24034465 PMC4053985

[8] Messerschmidt DM Knowles BB Solter D. DNA Methylation Dynamics During Epigenetic Reprogramming in the Germline and Preimplantation Embryos. Genes Dev (2014) 28:812–28. doi: 10.1101/gad.234294.113 PMC4003274 24736841

[9] Quach A Levine ME Tanaka T Lu AT Chen BH Ferrucci L. Epigenetic Clock Analysis of Diet, Exercise, Education, and Lifestyle Factors. Aging (Albany NY) (2017) 9:419–46. doi: 10.18632/aging.101168 PMC5361673 28198702

[10] Field AE Robertson NA Wang T Havas A Ideker T Adams PD. DNA Methylation Clocks in Aging: Categories, Causes, and Consequences. Mol Cell (2018) 71:882–95. doi: 10.1016/j.molcel.2018.08.008 PMC6520108 30241605

[11] Zhang W Qu J Liu GH Belmonte JCI. The Ageing Epigenome and its Rejuvenation. Nat Rev Mol Cell Biol (2020) 21:137–50. doi: 10.1038/s41580-019-0204-5 32020082

[12] Morrison MI Pither TL Fisher AJ. Pathophysiology and Classification of Primary Graft Dysfunction After Lung Transplantation. J Thorac Dis (2017) 9:4084–97. doi: 10.21037/jtd.2017.09.09 PMC5723840 29268419

[13] Cantu E Diamond JM Suzuki Y Lasky J Schaufler C Lim B. Quantitative Evidence for Revising the Definition of Primary Graft Dysfunction After Lung Transplant. Am J Respir Crit Care Med (2018) 197:235–43. doi: 10.1164/rccm.201706-1140OC PMC5768905 28872353

[14] Verleden GM Glanville AR Lease ED Fisher AJ Calabrese F Corris PA. Chronic Lung Allograft Dysfunction: Definition, Diagnostic Criteria, and Approaches to Treatment-A Consensus Report From the Pulmonary Council of the ISHLT. J Heart Lung Transplant (2019) 38:493–503. doi: 10.1016/j.healun.2019.03.009 30962148

[15] Calabrese DR Florez R Dewey K Hui C Torgerson D Chong T. Genotypes Associated With Tacrolimus Pharmacokinetics Impact Clinical Outcomes in Lung Transplant Recipients. Clin Transplant (2018) 32:e13332. doi: 10.1111/ctr.13332 29920787 PMC8103920

[16] Dugger DT Fung M Hays SR Singer JP Kleinhenz ME Leard LE. Chronic Lung Allograft Dysfunction Small Airways Reveal a Lymphocytic Inflammation Gene Signature. Am J Transplant (2021) 21:362–71. doi: 10.1111/ajt.16293 PMC8009189 32885581

[17] Muller F Scherer M Assenov Y Lutsik P Walter J Lengauer T. RnBeads 2.0: Comprehensive Analysis of DNA Methylation Data. Genome Biol (2019) 20:55. doi: 10.1186/s13059-019-1664-9 30871603 PMC6419383

[18] Subramanian A Tamayo P Mootha VK Mukherjee S Ebert BL Gillette MA. Gene Set Enrichment Analysis: A Knowledge-Based Approach for Interpreting Genome-Wide Expression Profiles. Proc Natl Acad Sci USA (2005) 102:15545–50. doi: 10.1073/pnas.0506580102 PMC1239896 16199517

[19] Dugger DT Fung M Zlock L Caldera S Sharp L Hays SR. Cystic Fibrosis Lung Transplant Recipients Have Suppressed Airway Interferon Responses During *Pseudomonas* Infection. Cell Rep Med (2020) 1:100055. doi: 10.1016/j.xcrm.2020.100055 32754722 PMC7402593

[20] Gordon ED Palandra J Wesolowska-Andersen A Ringel L Rios CL Lachowicz-Scroggins ME. IL1RL1 Asthma Risk Variants Regulate Airway Type 2 Inflammation. JCI Insight (2016) 1:e87871. doi: 10.1172/jci.insight.87871 27699235 PMC5033813

[21] Bonser LR Koh KD Johansson K Choksi SP Cheng D Liu L. Flow-Cytometric Analysis and Purification of Airway Epithelial-Cell Subsets. Am J Respir Cell Mol Biol (2021) 64:308–17. doi: 10.1165/rcmb.2020-0149MA PMC7909335 33196316

[22] Søraas A Matsuyama M de Lima M Wald D Buechner J Gedde-Dahl T. Epigenetic Age is a Cell-Intrinsic Property in Transplanted Human Hematopoietic Cells. Aging Cell (2019) 18:e12897. doi: 10.1111/acel.12897 30712319 PMC6413751

[23] Frobel J Rahmig S Franzen J Waskow C Wagner W. Epigenetic Aging of Human Hematopoietic Cells is Not Accelerated Upon Transplantation Into Mice. Clin Epigenet (2018) 10:67. doi: 10.1186/s13148-018-0499-7 PMC5964682 29796118

[24] Raddatz G Gao Q Bender S Jaenisch R Lyko F. Dnmt3a Protects Active Chromosome Domains Against Cancer-Associated Hypomethylation. PloS Genet (2012) 8:e1003146. doi: 10.1371/journal.pgen.1003146 23284304 PMC3527206

[25] Pasnupneti S Nicolls MR. Airway Hypoxia in Lung Transplantation. Curr Opin Physiol (2019) 7:21–6. doi: 10.1016/j.cophys.2018.12.002 PMC6688850 31403087

[26] Watson CJ Collier P Tea I Neary R Watson JA Robinson C. Hypoxia-Induced Epigenetic Modifications are Associated With Cardiac Tissue Fibrosis and the Development of a Myofibroblast-Like Phenotype. Hum Mol Genet (2014) 23:2176–88. doi: 10.1093/hmg/ddt614 24301681

[27] Tahiliani M Koh KP Shen Y Pastor WA Bandukwala H Brudno Y. Conversion of 5-Methylcytosine to 5-Hydroxymethylcytosine in Mammalian DNA by MLL Partner TET1. Science (2009) 324:930–5. doi: 10.1126/science.1170116 PMC2715015 19372391

[28] Thienpont B Steinbacher J Zhao H D'Anna F Kuchnio A Ploumakis A. Tumour Hypoxia Causes DNA Hypermethylation by Reducing TET Activity. Nature (2016) 537:63–8. doi: 10.1038/nature19081 PMC5133388 27533040

[29] Iasella CJ Hoji A Popescu I Wei J Snyder ME Zhang Y. Type-1 Immunity and Endogenous Immune Regulators Predominate in the Airway Transcriptome During Chronic Lung Allograft Dysfunction. Am J Transplant (2020) 21:2145–60. doi: 10.1111/ajt.16360 PMC8607839 33078555

[30] Sainani K. The Limitations of Statistical Adjustment. PM&R (2011) 3:868–72. doi: 10.1016/j.pmrj.2011.06.006 21944304

[31] Faust HE Golden JA Rajalingam R Wang AS Green G Hays SR. Short Lung Transplant Donor Telomere Length is Associated With Decreased CLAD-Free Survival. Thorax (2017) 72:1052–4. doi: 10.1136/thoraxjnl-2016-209897 PMC6550329 28446663

[32] Lu AT Xue L Salfati EL Chen BH Ferrucci L Levy D. GWAS of Epigenetic Aging Rates in Blood Reveals a Critical Role for TERT. Nat Commun (2018) 9:387. doi: 10.1038/s41467-017-02697-5 29374233 PMC5786029

[33] Heylen L Thienpont B Naesens M Lambrechts D Sprangers B. The Emerging Role of DNA Methylation in Kidney Transplantation: A Perspective. Am J Transplant (2016) 16:1070–8. doi: 10.1111/ajt.13585 26780242

[34] Huang N Tan L Xue Z Cang J Wang H. Reduction of DNA Hydroxymethylation in the Mouse Kidney Insulted by Ischemia Reperfusion. Biochem Biophys Res Commun (2012) 422:697–702. doi: 10.1016/j.bbrc.2012.05.061 22627137

[35] Pratt JR Parker MD Affleck LJ Corps C Hostert L Michalak E. Ischemic Epigenetics and the Transplanted Kidney. Transplant Proc (2006) 38:3344–6. doi: 10.1016/j.transproceed.2006.10.112 17175268

[36] Parker MD Chambers PA Lodge JP Pratt JR. Ischemia- Reperfusion Injury and its Influence on the Epigenetic Modification of the Donor Kidney Genome. Transplantation (2008) 86:1818–23. doi: 10.1097/TP.0b013e31818fe8f9 19104428

[37] Schaenman J Zhou X Guo R Rossetti M Liang EC Lum E. DNA Methylation Age Is More Closely Associated With Infection Risk Than Chronological Age in Kidney Transplant Recipients. Transplant Direct (2020) 6:e576. doi: 10.1097/TXD.0000000000001020 33134500 PMC7581059

